# Engine knock detection for a multifuel engine using engine block vibration with statistical approach

**DOI:** 10.1016/j.mex.2021.101583

**Published:** 2021-11-18

**Authors:** Muammar Mukhsin Ismail, Mas Fawzi, Juntakan Taweekun, Theerayut Leevijit

**Affiliations:** a Centre for Energy and Industrial Environment Studies (CEIES), Universiti Tun Hussein Onn Malaysia, Parit Raja, Johor 86400 Malaysia; b Energy Technology Program, Faculty of Engineering, Prince of Songkla University, Hat Yai, Songkhla 90112, Thailand

**Keywords:** Engine knock phenomena, Knock detection method, Diesel-CNG dual fuel

## Abstract

Engine knock is an obstacle for maximizing CNG fuel utilization on a Diesel-CNG Dual Fuel engine. Prolong experience of this phenomenon may lead to severe engine damage. The low intensity of this phenomenon is difficult to recognize due to other noises from the engine. Thus, improper engine tuning techniques may make this phenomenon unnoticeable until the engine damages. Knock phenomena on such engines may not be detected in the combustion analysis graph. Its random occurrence in consecutive engine cycles makes it difficult to be seen using visual data. This knowledge gap, if closed, can lead to an opportunity for knock avoidance on the multifuel engine. This work proposed a method to quantify the knock occurrence based on engine block vibration using a single piezoelectric knock sensor. The knock occurrence was detected by comparing the calculated knock index with the knock threshold, determined using a statistical three-sigma rule analysis. This method can index the knock intensity, detect the engine knock occurrence, and visualize the knock phenomenon.

• This paper describes an alternative engine knock detection technique based on engine block vibration.
• This method proposes the knock threshold determination based on statistical three-sigma rule analysis.
• This method is capable of visualizing the knock phenomenon in consecutive and at each engine cycle.

## Graphical abstract

**Figure:**
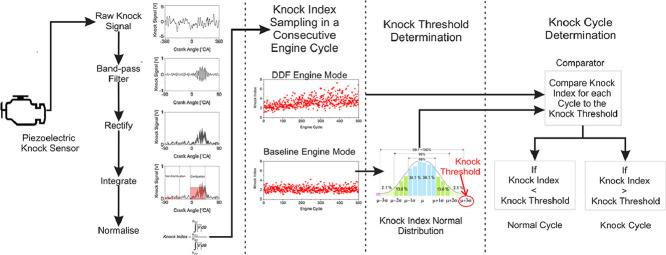

Specifications Table

**Table:** 

Subject Area:	Engineering
More specific subject area:	Automotive and Combustion
Method name:	Engine Knock Detection for a Multifuel Engine using Engine Block Vibration with Statistical Approach
Name and reference of original method:	Millo, F. and Ferraro, C., "Knock in S.I. engines: A comparison between different techniques for detection and control," SAE Technical Paper 982477, 1998, 10.4271/982477.
Resource availability:	*Mukhsin, Muammar (2021), “Knock Signal 1400 rpm”, Mendeley Data, V1, doi: 10.17632/jzmhw4mbn7.1*

## Method details

A Diesel-CNG Dual Fuel (DDF) engine utilizes CNG fuel on a dedicated diesel engine. It operates by supplying a portion of the CNG fuel inside the cylinder and ignited by a small portion of diesel fuel. However, engine knock occurs when CNG fuel quantity exceeds the limit [1], [2], [3], [4], [5], [6], [7], which is a part of the obstacle in establishing a DDF engine. Engine knock is a situation when the engine operates with annoying sounds such as ‘ticking’, ‘pinging’, or ‘clunking’ and is classified as abnormal combustion. Its continuous occurrence leads to severe engine damage such as piston surface erosion, head gasket failure, scuffing cylinder liner, spark plug damage, and valve melt [4,[8], [9], [10], [11].

Knock combustion on a DDF engine differs from a typical Spark Ignition (SI) and Compression Ignition (CI) engine. Since it comprises two types of fuel with different properties, CNG fuel pre-ignition, end-gas autoignition, rapid combustion, and late combustion were possible to occur. Unfortunately, these events may be undetected in the combustion graph due to several reasons, such as unsuitable frequency response of a pressure transducer [12], insufficient resolution of a data logger, signal conditioning of a data logger, energy release abruption during combustion, or improper combustion timing that leads to the peak of combustion process not occurs at the optimum moment. Several studies about the knock combustion on the DDF engine have been reported [4,[13], [14], [15], [16], [17], [18], but the presented result did not address this phenomenon. Yet, its occurrence at a random engine cycle makes it difficult to be traced from a sample set of combustion, and undoubtedly hard to be recorded, and too risky if using an optical engine.

Several knock detection methods were suggested to be used, such as using a sound level meter [19], in-cylinder pressure [20], [21], [22], accelerometer [23], and ion sensor [24]. A combination of a few methods was also suggested to improve knock detection [25], [26], [27], [28]. However, these methods were unworthy just to determine the knocking cycle for data extraction or engine tuning. Furthermore, an unsuitable knock threshold used may lead to false detection [29,30].

Thus, this work proposed an alternative knock detection method based on the integral of the absolute value of the first derivative of the band-pass filtered vibration signal with a statistical approach. The engine knock occurrence was quantified using a vibration signal from a piezoelectric knock sensor. The knock threshold was obtained using a statistical three-sigma rule analysis to determine the knock occurrence, which is an anomaly event on the diesel baseline engine. The continuity of this study is to develop a stand-alone engine knock metering unit for the DDF engine tuning and detecting the knock cycle for data extraction.

## Equipment setup

**Fig. 1:**
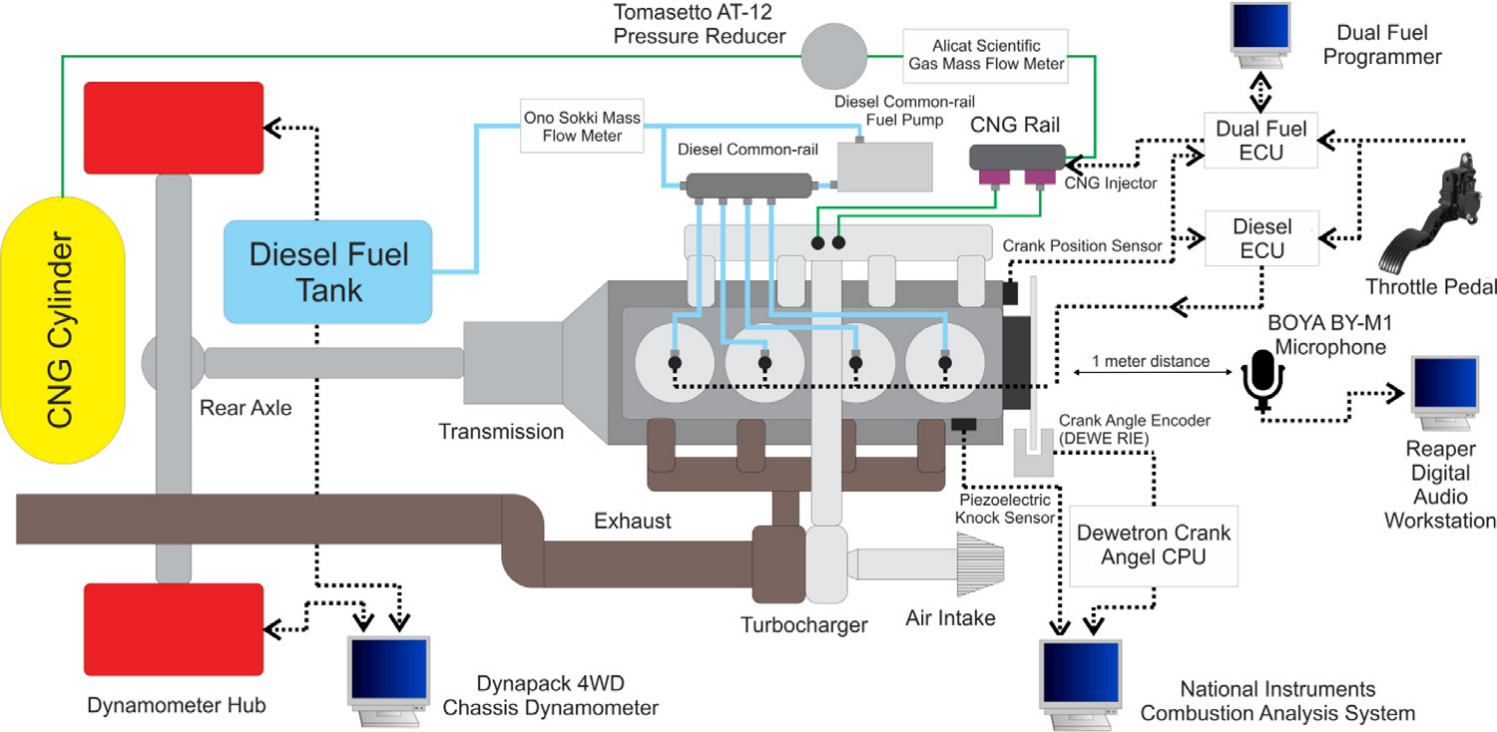
Equipment assembly diagram.

This work used a Toyota Hilux with a 2.5 liter common-rail direct injection diesel engine. The vehicle was attached to a Dynapack 4WD chassis dynamometer for engine braking to achieve a steady-state engine speed, as shown in Fig. 1. The engine vibration signal was obtained from a piezoelectric knock sensor manufactured by Continental VDO for automotive purposes. It was mounted near the first engine cylinder at the engine block to minimize any interference noise from another engine mechanism. The knock sensor signal was clocked based on crank angle timing using a Dewetron Crank Angle CPU and a Dewe-RIE crank angle encoder. A marker disk with 360 slits was mounted at the crank pulley to provide 1 °CA resolution for capturing the data. The signal from the knock sensor and crank angle encoder was recorded using a National Instruments Combustion Analysis System (NI-CAS).

The original diesel engine was converted to a DDF system by installing an additional gas fuel system comprising a CNG cylinder, Tomasetto AT-12 pressure reducer, CNG fuel injector, and a dual fuel ECU [44], [45], [46]. The original crank position sensor and throttle pedal were connected to both diesel and dual-fuel ECU for the DDF engine operation. The experiment was conducted by setting a desire engine speed on a Dynapack 4WD chassis dynamometer, and the throttle pedal was pressed to achieve the desired diesel fuel quantity. The diesel fuel quantity was measured using an Ono Sokki Mass Flow Meter installed at a low-pressure side between a diesel fuel tank and diesel common-rail fuel pump. In the DDF mode, CNG fuel quantity was adjusted using a dual fuel programmer. The CNG fuel quantity was measured using an Alicat Scientific Gas Mass flow meter installed at the low-pressure side between the Tomasetto AT-12 pressure reducer and the CNG injector. A BOYA BY-M1 omnidirectional microphone was placed 1 meter from the engine bay, and it was connected to Reaper Digital Audio Workstation to record environment audio. During the experiment, the environment audio was recorded with a 120 kHz sampling rate to confirm the observed knock occurrence.

## Knock index

**Fig. 2:**
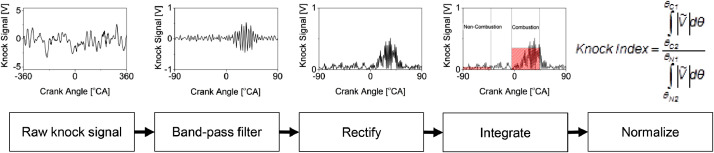
Knock index calculation process.

The knock index is a dimensionless unit to quantify the knock intensity for each engine cycle. It was determined using the integral of the absolute value of the first derivative of the band-pass filtered vibration signal [23]. This process comprises the band-pass filter, rectify, integrate, and normalize, as shown in Fig. 2.

**Fig. 3:**
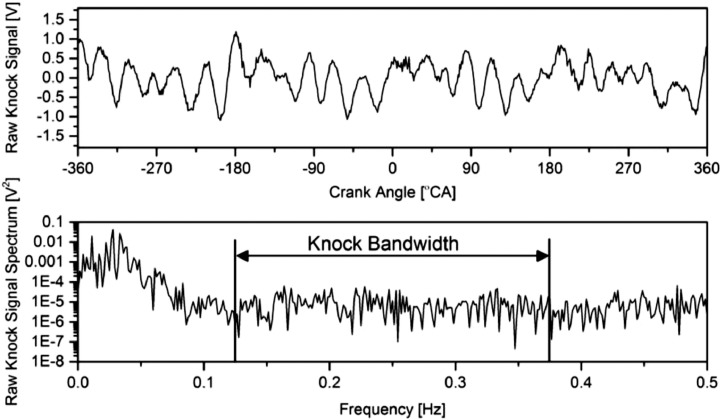
Raw knock signal and its spectrum.

Although the knock sensor was located near the engine cylinder, the raw knock signal contains some noises from several manners such as electrical signal noise, valve and camshaft mechanism, fuel injector, and natural engine vibration, as shown in Fig. 3. Thus, the raw knock signal in the crank angle domain was transformed into the frequency domain using Fast Fourier Transform (FFT) before band-pass filtered using second-order Butterworth to offset the waveform at *y*=0. Based on the literature, there is no specific standard for knock bandwidth [25,^31^,32]. It depends on the data acquisition resolution, sampling rate, and sampling domain, either time-domain or crank-angle-domain. Most of the studies had suggested that the knock bandwidth was estimated using Draper's equation with a Bessel's constant [23,^25^,33]. However, the estimated bandwidth was significantly high to 20,000 Hz and unsuitable for low-resolution sampling rate equipment.

Since the knock bandwidth is just an estimation, the band-pass filter can be done by trial and error calculation to eliminate undesired waveforms while keeping the distorted waveform. A signal processing program was used in this step because it is more practical and faster than manual calculation. This study used OriginPro software to determine the knock bandwidth at a 1 °CA sampling rate and found that the knock bandwidth was within 0.125 Hz to 0.375 Hz. Once the knock bandwidth is determined, it can be applied at any speed and load condition but is only valid for that engine.

**Fig. 4:**
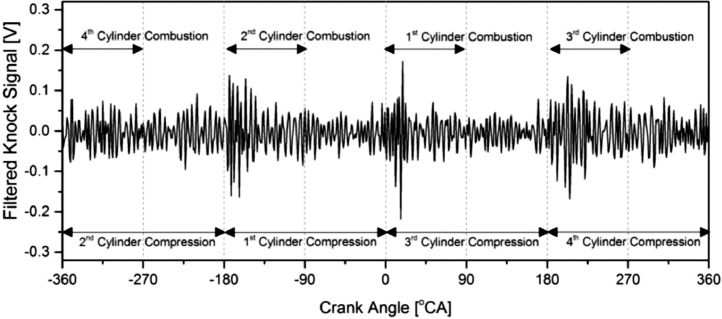
Filtered knock signal in 0.125 Hz to 0.375 Hz bandwidth.

The raw knock signal was band-pass filtered by cutting off the low frequency below 0.125 Hz and high frequency above 0.375 Hz. The band-pass filtered knock signal yields a graph as depicted in Fig. 4. Since the engine used was a four cylinders engine with 1–3–4–2 firing order, four combustion locations were determined within -360 °CA to -270 °CA for the fourth cylinder, -180 °CA to 90 °CA for the second cylinder, 0 °CA to 90 °CA for the first cylinder, and 180 °CA to 270 °CA for the third cylinder. According to the figure, a high knock signal amplitude was observed at the second, first, and third cylinders. The knock signal amplitude for the first engine cylinder was the highest because it closes to the knock sensor. When the cylinder location was farther from the knock sensor, the transverse wave from that cylinder was suppressed by another transverse wave from another source, leading to vibration energy dissipation before reaching the knock sensor. Hence, the knock signal amplitude for the fourth cylinder combustion was unable to be detected. Although the raw knock signal was filtered, some noises were observed during the compression stroke period, coming from another engine mechanism with a similar bandwidth range to the knock.

**Fig. 5:**
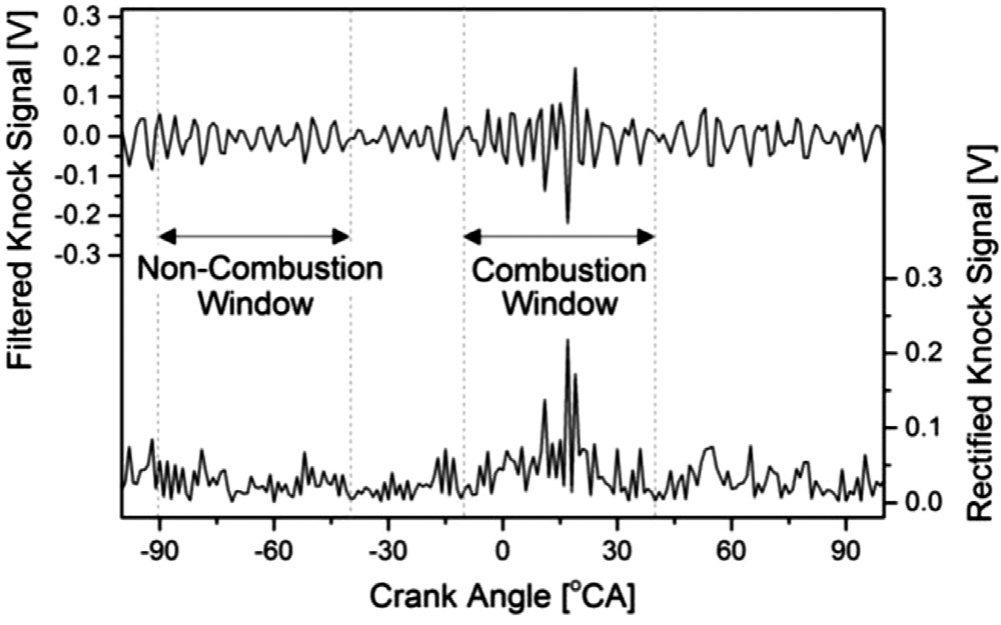
Selected location of combustion and non-combustion window.

A possible combustion window during the knock onset was selected. The original method suggested that the combustion window should start shortly after Top Dead Centre (TDC) to avoid piston slap noise; meanwhile, it should be ended at 50 °CA to cut off or minimize the valve closing noises [23]. However, it can be adjusted according to the purpose of the study since the knock combustion can occur in a wide range of crank angle degrees. Thus, this study chose the combustion window from -10 °CA to 40 °CA, as depicted in Fig. 5, because the diesel injection timing was before the TDC, and a DDF engine had a possibility to the knock onset due to pre-combustion.

Referring to Fig. 5, a low knock signal oscillation during the compression stroke indicates the background noise was present along with the crank angle. Thus, the knock signal was normalized by dividing the combustion window to the non-combustion window to obtain a signal-to-noise ratio comparable to every engine cycle. The non-combustion window should be located near the TDC to avoid overlapping knock signals from the second cylinder combustion. However, in this study, the non-combustion window was selected from -90 °CA to -40 °CA to avoid diesel pre-injection noise in between -40 °CA and -10 °CA. The selected knock signal at the combustion and non-combustion windows were rectified, integrated, and normalized using Eq. (1):

(1) $$\text{Knock}\;\text{Index}=\int_{\theta_{C2}}^{\theta_{C1}}\left|\tilde{V}\right|d\theta /\int_{\theta_{N2}}^{\theta_{N1}}\left|\tilde{V}\right|d\theta$$

Where *θ_C1_* is the crank angle corresponding to the start of combustion window, *θ_C2_* is the crank angle corresponding to the end of combustion window, *θ_N1_* is the crank angle corresponding to the start of the non-combustion window, *θ_N2_* is the crank angle corresponding to the end of the non-combustion window, and $\tilde{V}$ is the filtered knock signal.

## Knock threshold

The knock threshold was introduced to determine the knock index limit before the knock onset. It was obtained from the baseline engine, where all the settings were in the original condition. Since the engine was optimally set by the engine manufacturer, such as compression ratio and diesel injection timing, the knock phenomenon on the baseline engine is almost impossible to occur. The knock index from 500 consecutive engine cycles from the baseline engine was collected and plotted in a histogram. The number of consecutive engine cycles was optional. A small cycle number leads to miss capturing of the knock cycle; meanwhile, a large cycle number increases its margin of error. The histogram was plotted by considering its population mean, standard deviation, and optimum bin size using Eqs. 2–4 [34].

(2) $$\mu =\sum x_{\text{KI}}/N$$

(3) $$\sigma =\sqrt{\sum {\left(x_{\text{KI}}-\mu \right)}^{2}/\left(N-1\right)}$$

(4) $$\text{Bin}\;\text{Size}=3.49\sigma /\sqrt[{3}]{N}$$

Where *X_KI_* is the knock index, *µ* is the population mean, *σ* is the standard deviation, and *N* is the sample size.

**Fig. 6:**
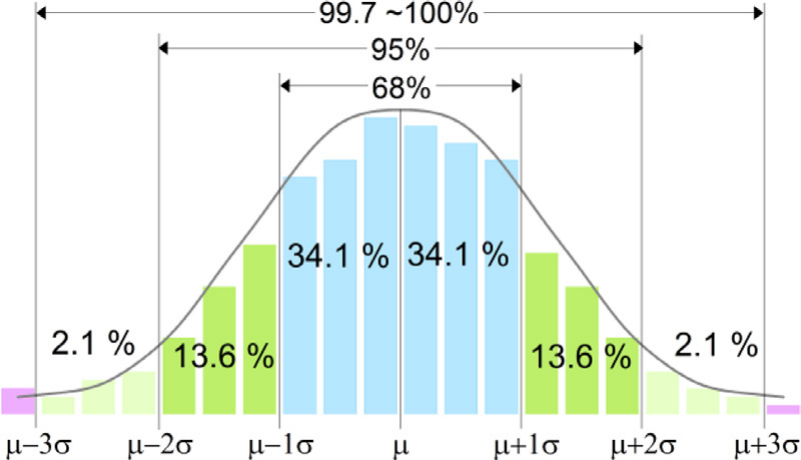
Approximated population data percentages in a normal distribution histogram.

The knock occurrence on the baseline engine was an anomaly event because it is almost impossible to occur. A three-sigma rule was applied to detect the anomaly event in a set of samples. The three-sigma rule explains that almost all the data in a normal distribution is distributed within a three standard deviation from the mean. As shown in Fig. 6, approximately 99.70 % of the data lies within three standard deviations, 95.00 % of the data lies within two standard deviations, and 68.00 % of data lies within one standard deviation from the mean. Data that lies outside of the three standard deviation range was assumed as irregular data, and its probability was at most 5.00 % [35].

The condition applied for the three-sigma rule was a unimodal normal distribution, as shown in Fig. 6. The normality test was conducted to ensure the knock index was normally distributed and yielded a bell-curve shape. The normal probability density function was calculated using Eq. 5. If the graph yields a bell-curve shape, the knock index was normally distributed and valid for the three-sigma rule analysis.

(5) $$\text{Normal}\;\text{Distribution}\;\text{Curve},y={\left(1/\sqrt[{\sigma }]{2\pi }\right)}^{e-{\left(x-\mu \right)}^{2}/{2_{\sigma }}^{3}}$$

Where *x* is the distance along the horizontal axis, and *y* is the distance along the vertical axis.

## Method validation

### Knock indexing and engine knock detection

An experiment was conducted with various diesel to CNG fuel ratios at constant 1400 rpm engine speed and 0.7 of equivalence ratio using the setup shown in Fig. 1. The tested fuel were 100D, 90D10G, 80D20G, 70D30G, and 60D40G represents diesel to CNG fuel mass flow rate 100:0, 90:10, 80:20, 70:30, and 60:40. In order to obtain a constant 0.7 equivalence ratio, the diesel and CNG fuel mass flow rate was calculated using the following equation.

(6) $$\varphi =AFR_{DDFstoi}/AFR_{DDF}$$

Where, *ϕ* is the equivalence ratio, *AFR_DDFstoi_* is the stoichiometric air-fuel ratio for DDF, and *AFR_DDF_* is the desired air-fuel ratio. Since the DDF mode comprises two fuel types, the *AFR_DDFstoi_* was calculated by summing up the stoichiometric air-fuel ratio of diesel and CNG based on its fraction, as shown in Eq. (7).

(7) $$AFR_{DDFstoi}=\left(z\%\times AFR_{dieselstoi}\right)+\left(100-z\%\times AFR_{CNGstoi}\right)$$

Where, *z%* is the percentages of diesel fuel mass fraction in the diesel-CNG fuel ratio, *AFR_dieselstoi_* is the stoichiometric air-fuel ratio for diesel, and *AFR_CNGstoi_* is the stoichiometric air-fuel ratio for CNG. When the *AFR_DDFstoi_* and *AFR_DDF_* were obtained, the desired fuel mass flow rate for DDF mode was calculated using Eq. (8).

(8) $$AFR_{DDF}={\dot{m}}_{air}/{\dot{m}}_{DDF}$$

Where, *ṁ_air_* is the measured air mass flow rate, and *ṁ_DDF_* is the desired total fuel mass flow rate. Hence, the global equation for this solution is interpreted in Eq. (9).

(9) $${\dot{m}}_{\text{DDF}}=\left[\left(\varphi \times {\dot{m}}_{\text{air}}\right)/\left(\left(z\%\times \text{AF}R_{\text{dieselstoi}}\right)+\left(100-z\%\times \text{AF}R_{\text{CNGstoi}}\right)\right)\right]$$

The desired diesel and CNG fuel mass flow rate were calculated using Eqs. (10) and (11).

(10) $${\dot{m}}_{\text{diesel}}=z\%\times {\dot{m}}_{\text{DDF}}$$

(11) $${\dot{m}}_{\text{CNG}}={\dot{m}}_{\text{DDF}}-{\dot{m}}_{\text{diesel}}$$

Where *ṁ_diesel_* is the diesel fuel mass flow rate, and *ṁ_CNG_* is the CNG fuel mass flow rate. Since this study using a CNG port injection method, the procedure from Eqs. (8) to (11) have to be repeated with trial and error to obtain both fuel mass flow rate at the desired equivalence ratio.From the experiment, the data recorded were the knock signal waveform for 500 consecutive engine cycles and 30 s of environment audio waveform with a 120 kHz sampling rate.

**Fig. 7:**
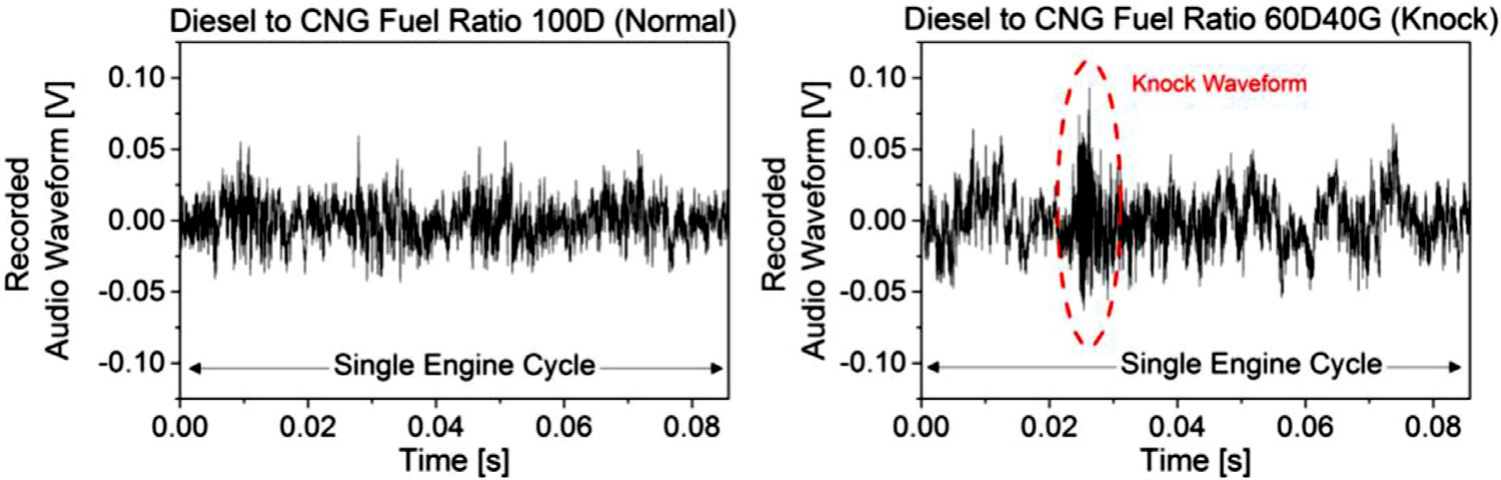
Recorded audio waveform between normal and knock engine operation.

According to the experiment observation by human hearing sense, the 100D, 90D10G, 80D20G, and 70D30G fuel ratios were determined as a normal operation; meanwhile, the 60D40G fuel ratio was determined as a knock operation. The recorded environment audio was used to confirm the result by observing the waveform pattern, as shown in the example in Fig. 7. According to the figure, a high waveform amplitude indicates the loudness of sound; meanwhile, a rapid amplitude fluctuation (either low or high) indicates the frequency of sound, which affects the sound pitch. This waveform contains a multi-frequency soundwave from various sources such as detonation from engine combustion, mechanical friction and vibration, wind noise from radiator fan, echo reflection in the engine bay, and others. A normal engine operation has a consistent sound intensity pattern in a consecutive engine cycle due to its combustion uniformity inside the cylinder. Although the waveform contains several noises, it yields an almost periodic pattern, as shown by the 100D fuel ratio. Meanwhile, a knock engine operation runs unsteadily with uncertain knocking sounds in a consecutive engine cycle. It yields an aperiodic waveform pattern with a rapid fluctuation. The knock waveform can be determined by a brief burst of amplitude which yields a sharp spike as shown by the 60D40G fuel ratio in Fig. 7.

**Fig. 8:**
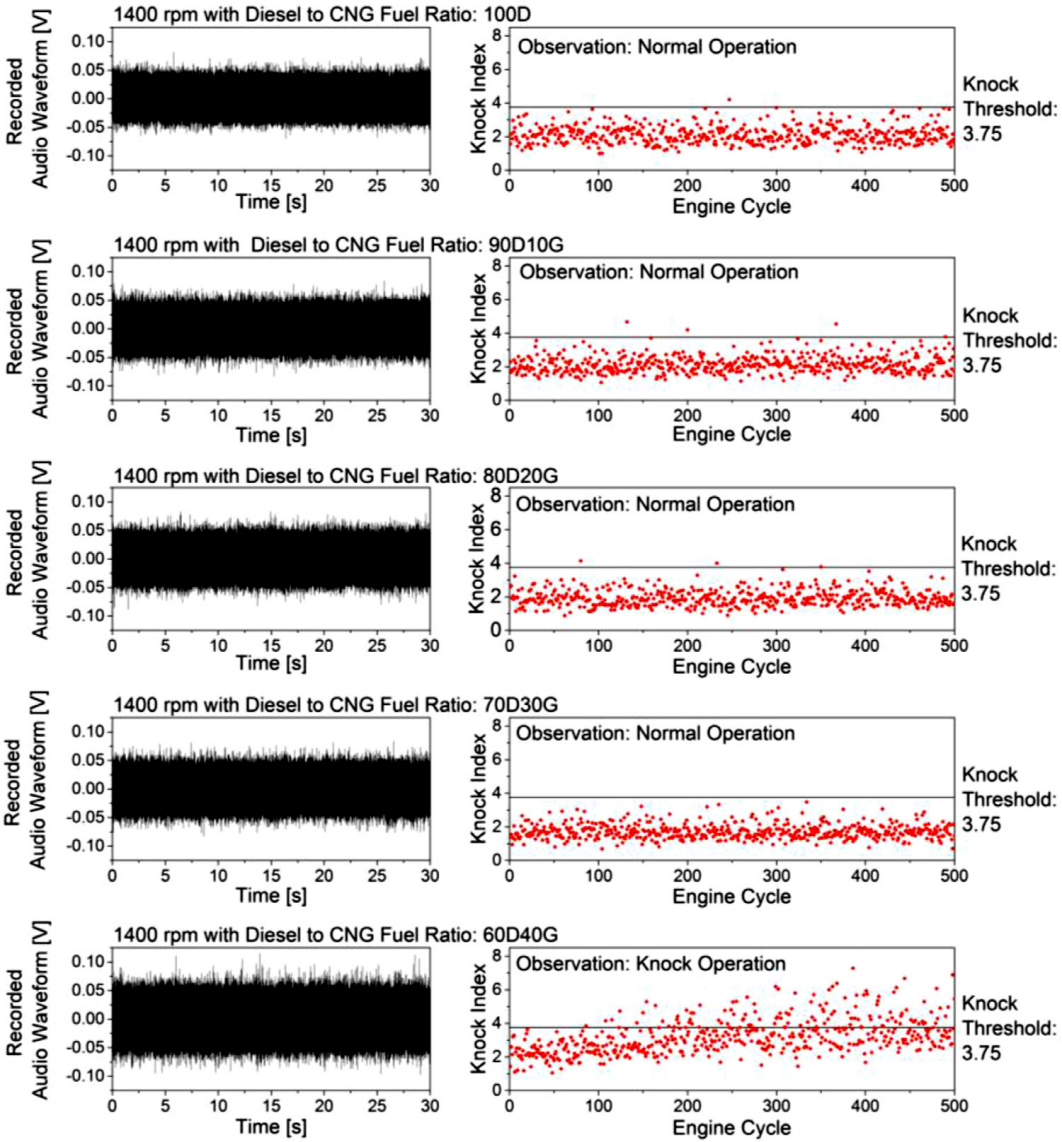
. Recorded audio waveform and knock index distribution for the 100D, 90D10G, 80D20G, 70D30G, and 60D40G fuel ratios.

Knock index for each engine cycle and fuel ratio were calculated using Eq. 1. The calculated knock index was compared to the knock threshold to determine the knock cycle, as shown in Fig. 8. According to the figure, most of the knock index for the 100D, 90D10G, 80D20G, and 70D30G fuel ratios were distributed below the knock threshold, with a few knock index lies at or just above the knock threshold line. It indicates that 90D10G, 80D20G, and 70D30G fuel ratios were in a regular engine operation similar to the 100D fuel ratio. A few knock index that lies just above the knock threshold may be caused by a high intensity of normal cycle or low intensity of knock cycle, which hard to notice during the engine operation. Although it was harmless to the engine, it was assumed as a knock cycle since it is significantly far from the sample mean. Thus, the unnoticeable event can be visualized by the knock index distribution.

The 60D40G fuel ratio was determined as a knock operation during the experiment. According to the observation, the engine knock did not promptly occur at the beginning of the experiment. Unstable engine operation was observed soon after and followed by an aperiodic knocking sound afterward. The knock occurred due to heat-accumulating inside the cylinder and led the CNG fuel was gradually preheated against the engine cycle increment. As reported by several works of literature, the increase of CNG fuel temperature increases the knock occurrence tendency [4,^15^,36]. Recorded audio waveform could not interpret this phenomenon because it contains vibration noise due to unstable engine operation. It can only show the knock occurrence by a high amplitude waveform beyond the range of -0.05–0.05 V. In agreement with the observation, the knock index distribution for the 60D40G fuel ratio was lower than the knock threshold at the beginning of the engine cycle. After several engine cycles, the knock index distribution was dispersed with an increment trend above the knock threshold. In this study, the characteristic of the knock phenomenon in a consecutive engine cycle can be visualized by the knock index distribution and agrees with the experimental observation.

### Data selection from the engine cycle

**Fig. 9:**
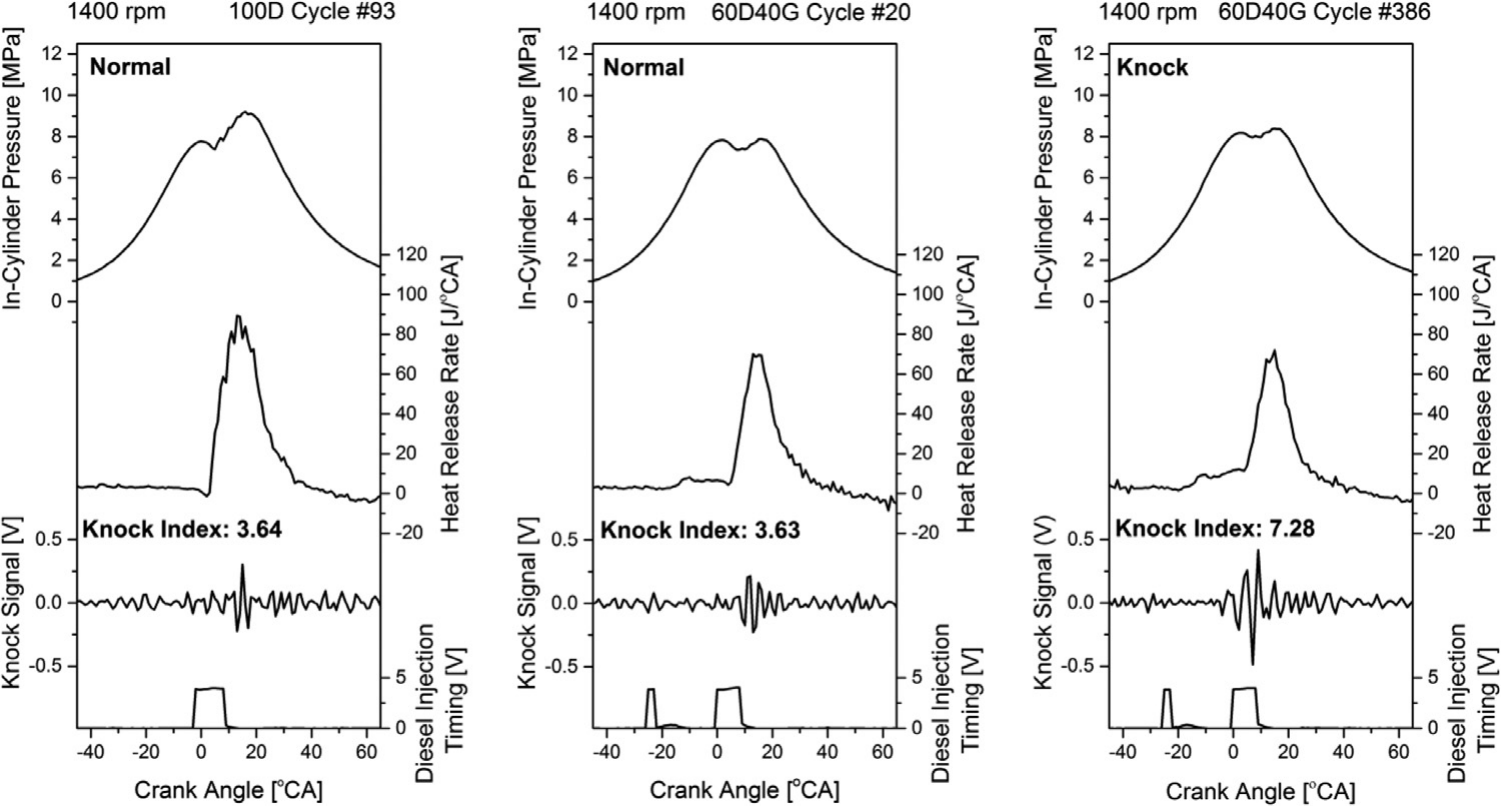
Selected combustion data and knock signal for the 100D and 60D40G fuel ratio at 1400 rpm engine speed.

The knock phenomenon on a DDF engine is rarely can be detected in the combustion analysis graph. However, it is vitally important to determine the knock combustion at the specific engine cycle on the DDF engine. Such information may be crucial to saving engine damages and losses caused by the knock phenomenon. This proposed method simplified the tracing process by selecting the engine cycle to form the knock index distribution graph. For example, according to the knock index distribution graph in Fig. 8, the 93^rd^ engine cycle for the 100D fuel ratio and the 20^th^ engine cycle for the 60D40G fuel ratio was selected due to its knock index closest to the knock threshold. The knock index of the 386^th^ engine cycle for the 60D40G fuel ratio was selected due to the highest knock index, which is expected to be the knock combustion. The combustion data from the selected engine cycle was post-processed for further analysis, as depicted in Fig. 9. It is known that the energy released during the combustion yields vibration and resulting in the knock signal oscillation. Therefore, a hypothesis can be made to determine the probable causes of the knock occurrence by comparing the knock signal and heat release rate.

The 100D fuel ratio at the 93^rd^ engine cycle and 60D40G fuel ratio at the 20^th^ engine cycle were normal combustions. The maximum knock signal amplitude was observed during the heat release rate increment and possibly due to the shockwave collision during the flame propagation. Since the conducted experiment was to test the proposed method, all the diesel ECU settings were in default as set by the engine manufacturer (Denso Corporation). At a low-speed and low-load, the second generation diesel common-rail used on this system introduces diesel pre-injection at a few°CA before the main injection to reduce noise and emissions [37], [38], [39], [40], [41], [42], [43]. Unlike the 100D, a reduction of diesel fuel quantity at the 60D40G fuel ratio consequently reduces the engine load as calculated by the ECU and leads the diesel pre-injection occurring at the -26 °CA. However, the introduced diesel pre-injection at this fuel ratio did not promptly lead to the knock phenomenon. After several engine cycles, the CNG fuel temperature increases and leads the combustion to occur before the TDC, as shown by the 386^th^ engine cycle. The opposition of combustion pressure with the piston movement yields a shockwave collision and possibly leads to the knock occurrence.

**Fig. 10:**
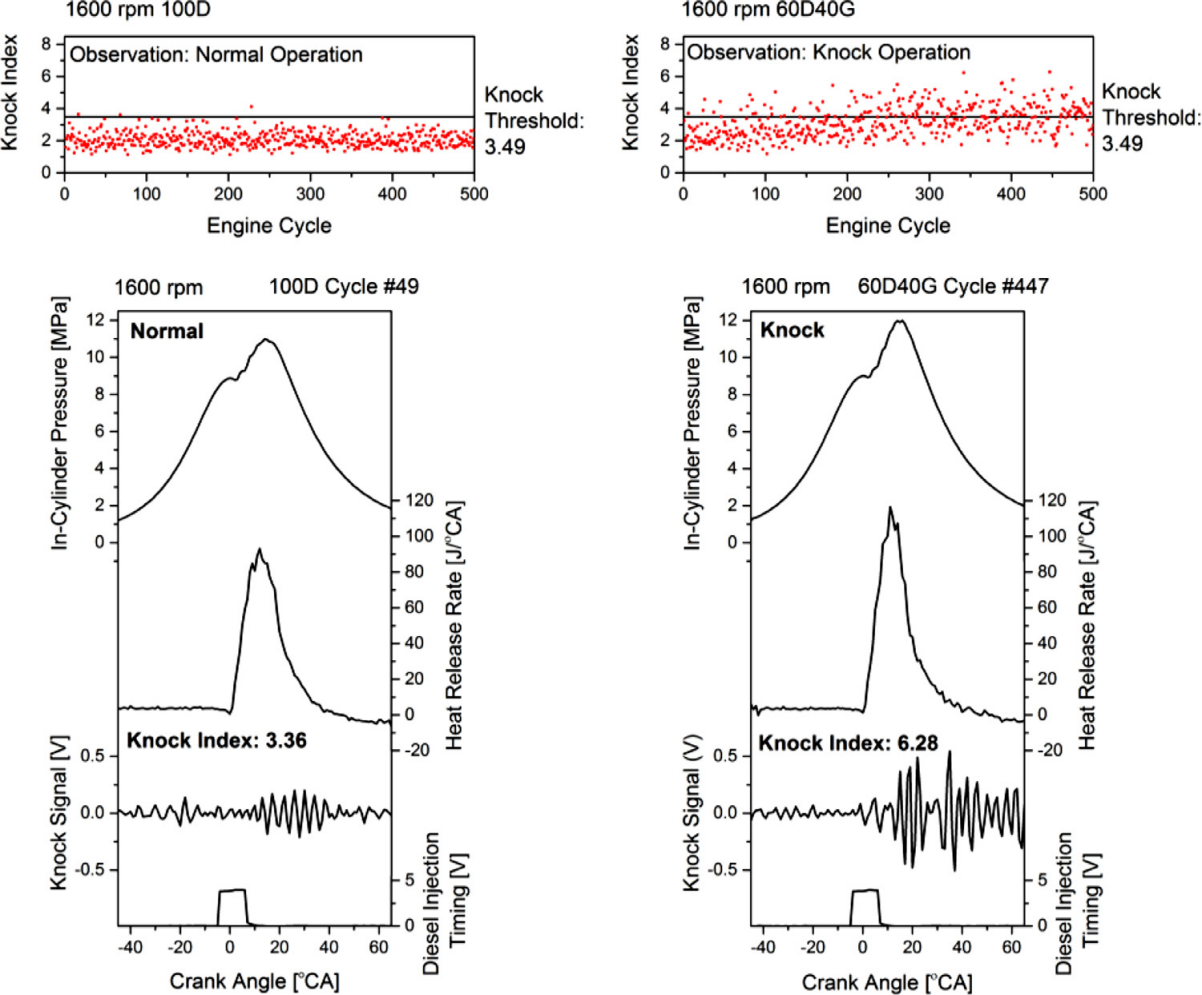
Selected combustion data and knock signal for the 100D and 60D40G fuel ratio at 1600 rpm engine speed.

Another experiment was conducted at 1600 rpm engine speed using a constant diesel injection timing to show how the knock phenomenon occurs without diesel pre-injection. In this experiment, the 100D and 60D40G fuel ratio was tested, and the result is depicted in Fig. 10. The combustion data was selected from the 100D fuel ratio with the knock index closest to the knock threshold and the 60D40G fuel ratio with the highest knock index. Looking at the figure alone, the in-cylinder pressure and heat release rate graph for the 60D40G fuel ratio did not show any obvious sign of the knock occurrence. Even though the peak of in-cylinder pressure and heat release rate for the 60D40G fuel ratio was higher than the 100D fuel ratio, such evidence was not sufficient and is arguable to prove the knock occurrence. A rapid heat release rate for the 60D40G fuel ratio itself does not indicate the intensity of knock occurrence. However, the method proposed in this paper provides a solution to prove the knock occurrence. As an example, by filtering the knock signal, the cause of the knock phenomenon for the 60D40G fuel ratio can be hypothesized as an abruption of energy released and late combustion occurrence. Such evidence was presented by a high amplitude of the knock signal at the mixing-controlled combustion phase and late combustion phase. The rapid heat release rate showed abruption of energy released during the mixing-controlled combustion phase; meanwhile, the late combustion occurrence was shown by the heat release rate fluctuation during the late combustion phase.

Interestingly, the abruption of the heat release rate in late combustion shown in Fig. 10 (1600 rpm) at 60D40G is also observed in Fig. 9 (60D40G, #386). In Fig. 9 (1400 rpm), the knock signal is prominent in the initial combustion, and the knock signal is smaller in the late combustion where the abruption occurs. Whereas, in Fig. 10, the knock signal is more significant during late combustion. As described earlier, the engine at 1400 rpm and 1600 rpm have different injection strategies dictated by the stock engine ECU by estimating the engine load. At 1400 rpm with 60D40G, the ECU introduces a diesel pre-injection. This pre-injection plus the added CNG during intake causes the fuel-air mixture in the cylinder to reach a combustible condition during the compression stroke before reaching TDC. During compression stroke before TDC, the rapid gas expansion creates an opposing force to the raising piston, translating into knocking. Therefore, in Fig. 9 (60D40G, #386), we can observe an increase in heat release rate before TDC before the main diesel injection.

On the other hand, the engine at 1600 rpm (Fig. 10, 60D40G, #447) applies a single injection strategy that starts at about -5 °CA. In this case, the knock occurs during the mixing-controlled and late combustion phases. The possible reason is that a high amount of CNG added potentially yields a homogeneous fuel-air mixture that scatters distributed in the cylinder. After the diesel fuel ignition, some portion of CNG located near the diesel fuel front flame was combusted. The high reactivity of CNG leads to rapid energy releases at once and yields mechanical shock during the mixing-controlled combustion phase. In the next combustion phase, the diesel fuel diffusion flame still propagates and ignites the remaining unburned CNG fuel (outside the mixing-controlled flame area). It yields secondary active combustion and resulting in knocking during the late combustion phase. This secondary active combustion occurs with a low reaction rate and can be seen by a slight fluctuation of the heat release rate between 30 °CA to 50 °CA. Although this heat release rate fluctuation could not indicate the knock phenomenon, this proposed method is able to detect its occurrence using the filtered knock signal.

## Conclusion

The method detailed in this work enabled the quantification of any knock occurrence on a DDF engine. The intensity of the knocking sound for each engine cycle can be presented in terms of a knock index. The knock indexing method using the integral of the absolute value of the first derivative of the band-pass filtered vibration signal was capable to visually capture the knock phenomenon intensity in a consecutive engine cycle. The statistical three-sigma rule can be used to determine the knock threshold, which was suggested to be a boundary between normal and knock engine cycle using a diesel engine as a baseline.

This method was validated by several experiments using diesel to CNG fuel ratio on a DDF engine. The result showed that the knock index characteristic in a consecutive engine cycle was identical to the experimental observation. The engine cycle condition, either normal or knock, can be determined by comparing the knock index for each engine cycle with the knock threshold. Although the knock phenomenon on the DDF engine is a random occurrence, this method is capable of detecting the knock combustion at each engine cycle. Thus, the knock occurrence's possible causes can be further analyzed using the knock signal and combustion data at any engine cycle.

## Declaration of Competing Interest

The authors declare that there are no conflicts of interest in this work.
